# Thermochemical Valorization of Silkworm Feeding Waste: Chemical Characterization and Biological Activities of the Pyrolysis-Derived Liquid Fraction

**DOI:** 10.3390/antiox15091213

**Published:** 2026-09-21

**Authors:** Halil Karan, Aybeniz Yıldırım, Fırat Yılmaz, İnanç Özgen, Ercan Aydoğmuş, Abdulkadir Gül

**Affiliations:** 1 Department of Parks and Garden Plants, Keban Vocational School, Fırat University, Elazığ 23119, Turkey; 2 Department of Pharmacognosy, Faculty of Pharmacy, Fırat University, Elazığ 32119, Turkey; 3 Department of Food Engineering, Faculty of Engineering and Natural Sciences, Gumushane University, Gümüşhane 29100, Turkey; 4 Department of Bioengineering, Faculty of Engineering, Fırat University, Elazığ 23119, Turkey; 5 Department of Chemical Engineering, Faculty of Engineering, Fırat University, Elazığ 23119, Turkey; 6 Department of Emergency Aid and Disaster Management, Faculty of Health Sciences, Gumushane University, Gümüşhane 29100, Turkey

**Keywords:** silkworm feeding waste, pyrolysis liquid, chemical profile, antibacterial activity, antioxidant activity, anti-inflammatory activity, antidiabetic activity

## Abstract

Silkworm-rearing residues are an underused biomass resource that can be converted into value-added products. In this study, a condensable liquid product (SWP-Liq) was produced from these residues by pyrolysis at 325 ± 5 °C and evaluated for its chemical composition and in vitro biological activities. SWP-Liq yielded phenolic-, flavonoid-, and triterpenoid-equivalent values of 218.47 ± 22.40 mg GAE/g, 79.13 ± 3.55 mg QE/g, and 55.54 ± 6.15 mg EE/g, respectively. The ABTS•^+^ assay showed a higher radical-scavenging response than DPPH, while Fe^3+^- and Cu^2+^-reducing activities were also observed. SWP-Liq inhibited 5-LOX with an IC_50_ of 33.05 ± 1.10 µg/mL, whereas α-glucosidase inhibition was weaker. Antibacterial activity against all four tested bacterial species was observed at 8 mg/mL but not at 4 mg/mL. Targeted LC–MS/MS quantified five phenolic constituents, with *p*-coumaric acid (1599.71 µg/g), chlorogenic acid (871.43 µg/g), and ferulic acid (285.50 µg/g) being the most abundant. Overall, these findings support the further investigation of silkworm feeding waste as a feedstock for thermochemical valorization into chemically and biologically evaluable bio-based fractions.

## 1. Introduction

Agricultural, animal-derived, and agro-industrial wastes represent an important challenge for sustainable waste management but are increasingly recognized as renewable feedstocks for the production of value-added bio-based products [1,2,3,4]. In this context, the conversion of low-value biomass into chemically complex liquid fractions with potential biological properties has attracted increasing interest within circular-economy and waste-valorization strategies [5,6,7,8].

Sericulture is an economically important production sector primarily based on the rearing of *Bombyx mori* for silk production [9]. Because silkworms are predominantly fed mulberry (*Morus* spp.) leaves, their rearing generates substantial organic residues, including residual leaves, feeding waste, feces, and larval and pupal by-products [10,11]. Mulberry leaves contain phenolics, flavonoids, alkaloids, organic acids, and other secondary metabolites; therefore, silkworm feeding waste represents a chemically complex biomass containing both plant- and animal-derived constituents [9,11].

By-products associated with silkworm rearing and silk production have been explored for various applications. Sericulture by-products, particularly silk fibroin, sericin, pupae, and larval materials, have been extensively investigated for biomaterial, food, feed, and nutraceutical applications [12,13,14,15]. In contrast, feeding residues and fecal waste have mainly been considered for low-value applications such as composting, fertilizers, soil amendments, and feed, while their thermochemical conversion into chemically and biologically evaluable liquid products remains comparatively underexplored.

Conventional recovery of bioactive compounds from biomass commonly relies on solvent-based extraction techniques, which may involve substantial solvent consumption, prolonged processing, and subsequent solvent-removal steps [16,17]. Pyrolysis provides an alternative thermochemical valorization approach in which biomass is converted under an inert atmosphere into liquid, solid, and gaseous products [18,19]. This approach converts complex waste biomass into multiple potentially valuable product fractions without external organic extraction solvents [20,21].

Studies on waste-derived extracts and thermochemically generated liquid fractions have demonstrated measurable biological responses depending on their chemical composition and production conditions. Silkworm-derived materials, including protein hydrolysates and cocoon extracts, have been investigated for antioxidant and antibacterial properties, while larval by-products and other sericulture-related products have been evaluated for their effects on inflammation-related pathways and their antidiabetic potential [7,8,14,15,22]. Similarly, researchers have investigated pyrolysis liquids from agricultural wastes as chemically complex products in the context of green chemistry, sustainable agriculture, and biological activity screening [6,23]. However, research on silkworm feeding waste remains limited, particularly regarding its thermochemical conversion and the integrated chemical and biological characterization of the resulting pyrolysis-derived material.

The central hypothesis of this study was that silkworm feeding waste could be converted through pyrolysis into a chemically characterizable fraction exhibiting measurable in vitro biological responses. Complementary assays were selected to provide an initial functional characterization of this chemically complex pyrolysis-derived material: antioxidant assays were used to assess different aspects of redox behavior, 5-LOX and α-glucosidase inhibition were evaluated as enzyme-based responses related to leukotriene biosynthesis and carbohydrate digestion, respectively, and antibacterial screening was performed to assess growth-inhibitory effects against selected bacterial strains. Chemical characterization included total phenolic, flavonoid, and triterpenoid-equivalent determinations together with targeted LC–MS/MS profiling. The novelty of the present study lies not in the development of new analytical or biological methodologies, but in the integrated chemical and biological evaluation of a pyrolysis-derived liquid fraction obtained specifically from silkworm feeding waste, a comparatively underexplored sericulture residue. To the best of our knowledge, studies combining targeted phenolic profiling with antioxidant, 5-LOX-inhibitory, α-glucosidase-inhibitory, and antibacterial assessments of this specific pyrolysis-derived matrix remain scarce. Accordingly, this study aimed to evaluate the potential of pyrolysis as a valorization strategy for converting low-value silkworm feeding waste into a chemically and biologically evaluable bio-based liquid fraction by addressing three primary research questions: (i) whether the resulting fraction could be chemically characterized in terms of its major phytochemical groups and targeted phenolic constituents; (ii) whether it exhibited measurable antioxidant and enzyme-inhibitory responses; and (iii) whether it showed antibacterial activity against the selected bacterial strains.

## 2. Materials and Methods

### 2.1. Materials

Reagents and solvents required for the experimental procedures were purchased at analytical-grade purity from Merck KGaA (Darmstadt, Germany) or Sigma-Aldrich (St. Louis, MO, USA). The bacterial panel employed for antimicrobial testing comprised four ATCC strains (American Type Culture Collection, Manassas, VA, USA): *E. coli* 25922, *P. aeruginosa* 27853, *E. faecalis* 29212, and *S. aureus* 25923. These microorganisms were provided by the culture repository of the Microbiology Laboratory at Gümüşhane University.

### 2.2. Pyrolysis-Based Production of a Liquid Product from Silkworm Feeding Waste

**Figure 1:**
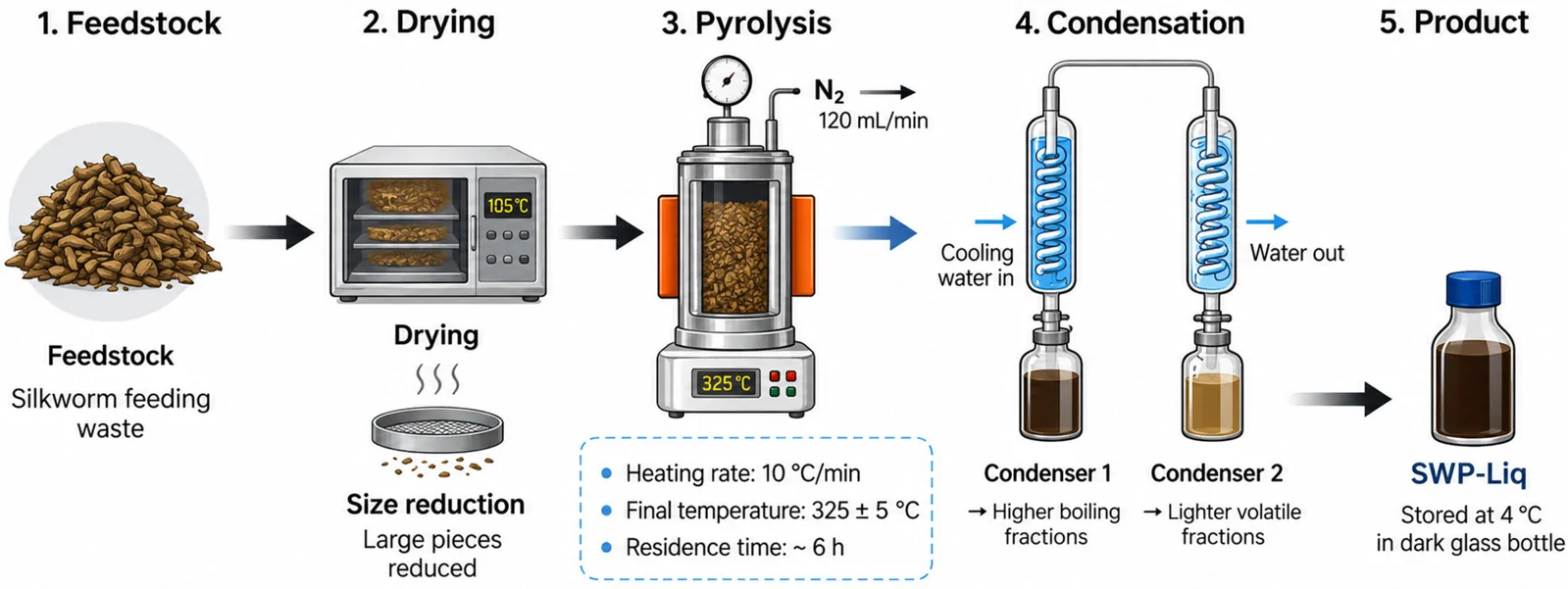
Schematic illustration of the pyrolysis-based procedure used to obtain the liquid fraction (SWP-Liq) from silkworm feeding waste.

SWP-Liq was generated by adapting the pyrolytic approach previously reported by Özgen et al. [24], with the complete sequence of experimental operations illustrated in Figure 1. The biomass used as pyrolysis feedstock originated from silkworms maintained in the laboratory for nearly two months between September and October 2025. Fresh mulberry leaves constituted the sole feed during this period. Rearing conditions were controlled at 25 ± 2 °C and a relative humidity of 70 ± 5%. Fecal matter together with uneaten feeding residues was periodically recovered during rearing and subsequently pooled to constitute the starting material for thermochemical processing.

A batch of approximately 2.0 kg of the collected material was initially passed through a sieve to eliminate dust and extraneous inorganic particles. No chemical treatment was applied before pyrolysis, thereby avoiding chemically induced alteration of the original feedstock. Coarse particles were mechanically reduced in size to improve material uniformity and promote more efficient thermal transfer. Moisture was subsequently removed by placing the prepared biomass in an oven (ThermoStable IG-105, DAIHAN Scientific, Wonju, Republic of Korea) at 105 °C for 24 h. This drying step reduced the initial feedstock mass from approximately 2.0 to 1.6 kg.

Thermochemical conversion was performed in a bench-scale fixed-bed pyrolysis unit equipped with proportional–integral–derivative (PID) temperature regulation. Heat losses from the stainless-steel reaction chamber were restricted by external insulation, while volatile products leaving the reactor were routed through two condensers arranged sequentially. An oxygen-limited environment was established by continuously supplying N_2_ at 120 mL/min throughout the process. The dried biomass was heated at 10 °C/min until the reactor temperature reached 325 ± 5 °C. Once this temperature range had been attained, thermal treatment continued for approximately 6 h, allowing prolonged decomposition of the feedstock and release of volatile pyrolysis products. The selected temperature falls within the low-temperature pyrolysis range reported for biomass conversion, in which both pyrolysis temperature and residence time are important determinants of thermochemical conversion and product characteristics [25,26]. Accordingly, the process employed in the present study differs from conventional fast pyrolysis and is more appropriately described as a low-temperature, prolonged fixed-bed pyrolysis treatment.

Vapors exiting the reactor were separated according to their condensation behavior. The first stage, cooled by circulating water, recovered the less volatile, higher-boiling constituents, whereas an ice-cooled second stage trapped the remaining lighter condensable fraction. Gaseous products that remained uncondensed were released through a dedicated exhaust fitted with a pressure-relief safety valve. Liquid recovered from the condensation stages was collected in glass containers. Processing approximately 1.6 kg of dried biomass under these conditions yielded about 200 mL of condensate. On a dry-feedstock basis, this represents a volumetric liquid yield of 0.125 mL/g, equivalent to 125 mL/kg.

The amount of nonvolatile residue contained in SWP-Liq was established by gravimetric measurement. An accurately measured 5-mL portion of the liquid was introduced into a glass weighing vessel that had previously been dried and weighed. The sample was then maintained at 105 °C until successive weighing indicated no further change in mass. Before the final weighing, the vessel was allowed to equilibrate to room temperature inside a desiccator. Subtraction of the initial vessel mass from its final mass after removal of the volatile fraction indicated approximately 50 mg of residual solids in the 5-mL aliquot. Accordingly, the concentration expressed on a dry-residue basis was approximately 10.0 mg/mL and was obtained from Equation (1):

(1) $$C=\frac{m_{2}-m_{1}}{V}$$

where C denotes the dry-residue concentration of SWP-Liq (mg/mL), *m*_1_ represents the mass of the dried empty vessel (mg), *m*_2_ corresponds to the final mass of the vessel containing the residual solids (mg), and *V* is the volume of SWP-Liq subjected to gravimetric determination (mL).

For subsequent chemical and biological analyses, SWP-Liq was evaporated to dryness, and the resulting nonvolatile residue was accurately weighed and reconstituted in the solvent appropriate for each assay to prepare the required test concentrations. Therefore, all concentrations reported in mg/mL or µg/mL for these analyses refer to the mass concentration of the reconstituted SWP-Liq dry residue. The value of approximately 10.0 mg/mL reported above refers solely to the dry-residue content of the original liquid product and does not represent the test concentration used in the individual assays.

### 2.3. Determination of Total Phenolic Content (TPC)

The phenolic-equivalent response of SWP-Liq was quantified by adapting the Folin–Ciocalteu assay of Kasangana et al. [27]. For each determination, 300 µL of SWP-Liq working solution (1 mg/mL) was combined with 3.4 mL deionized water and 0.5 mL methanol, followed by the introduction of 200 µL Folin–Ciocalteu reagent. The resulting solution was vortex-homogenized and kept for 10 min under ambient conditions before adding 600 µL Na_2_CO_3_ (10%, *w*/*v*). Following a second vortexing step, color development proceeded for 120 min at room temperature with protection from light. Optical density was subsequently recorded at 760 nm on a UV–Vis spectrophotometer (UV-1800, Shimadzu, Kyoto, Japan). A reagent blank, in which an equal volume of deionized water substituted for SWP-Liq, was processed in parallel. Quantification was performed against a gallic acid (GA) standard curve covering 10–250 µg/mL (y = 0.0057x + 0.0105; R^2^ = 0.9999), and the resulting TPC was expressed as milligrams of GA equivalents per gram of sample (mg GAE/g).

### 2.4. Determination of Total Flavonoid Content (TFC)

Flavonoid-equivalent content in SWP-Liq was quantified using an adapted colorimetric protocol derived from Kasangana et al. [27]. Briefly, a 500-µL portion of the SWP-Liq working solution (1 mg/mL) was combined with 3.2 mL methanol (30%, *v*/*v*), and the reaction was initiated by introducing 150 µL NaNO_2_ (0.5 M). After 5 min at ambient temperature, 150 µL AlCl_3_ (0.3 M) was incorporated into the reaction medium, which was then left for a further 5 min. The final alkalization step consisted of adding 1.0 mL NaOH (1 M). The resulting mixture was vortex-homogenized and maintained for 10 min at room temperature before spectrophotometric reading at 506 nm. Background absorbance was accounted for using a reagent blank prepared with 500 µL distilled water in place of SWP-Liq and processed through the same reaction sequence. Quercetin (Q) standards spanning 10–250 µg/mL provided the calibration relationship (y = 0.00019x − 0.00020; R^2^ = 0.9990) used for quantification. TFC was subsequently calculated and expressed as milligrams of Q equivalents per gram of sample (mg QE/g).

### 2.5. Determination of Total Triterpenoid Content (TTC)

The triterpenoid-equivalent content of SWP-Liq was quantified through an adapted vanillin–perchloric acid colorimetric assay based on Gülmez et al. [28]. The reaction was assembled directly in microplate wells by combining 10 µL of SWP-Liq with 15 µL of vanillin prepared in glacial acetic acid (5%, *w*/*v*) and 50 µL of perchloric acid. Color development was promoted by maintaining the plate at 60 °C for 45 min, after which the reaction was rapidly brought to ambient temperature using an ice–water bath. Each well was subsequently supplemented with 225 µL glacial acetic acid, and the optical response was measured at 548 nm. Escin (E), prepared over a concentration range of 1.75–250 µg/mL, was used to establish the calibration relationship (y = 0.00112583x + 0.0808207; R^2^ = 0.98). The TTC of SWP-Liq was calculated from this equation and expressed as milligrams of E equivalents per gram of sample (mg EE/g).

### 2.6. Determination of the Phenolic Profile of SWP-Liq by LC–MS/MS

The profiling and measurement of polyphenolic elements within the SWP-Liq extract were conducted via an LC-MS/MS setup: a Thermo Scientific Dionex Ultimate 3000 UHPLC system coupled to a tandem mass spectrometer equipped with an H-ESI ionization source (Thermo Fisher Scientific, Waltham, MA, USA). Before evaluation, the SWP-Liq specimen (initial solution at 10 mg/mL) was thinned with an appropriate diluent and filtered through a 0.22 µm PTFE membrane to make it ready for LC-MS/MS assessment. The evaluations followed the protocols recommended [24,29]. Substance recognition was achieved by aligning retention durations and distinct precursor-to-product ion changes with reference materials. Measurements were executed using calibration plots developed for individual reference compounds, with outcomes stated as extract concentrations (µg/g pyrolysis liquid). Data acquisition and processing were performed using Xcalibur software, version 2.2 (Thermo Fisher Scientific, Waltham, MA, USA).

### 2.7. Antioxidant Analyses

#### 2.7.1. Determination of DPPH Radical-Scavenging Activity

The ability of SWP-Liq to quench DPPH radicals was assessed by adapting the spectrophotometric protocol of Ahmed et al. [30]. A methanolic DPPH solution (0.1 mM) was prepared, and its initial optical density at 517 nm was measured using a Multiskan SkyHigh microplate spectrophotometer (Thermo Fisher Scientific, Waltham, MA, USA). was standardized to 0.98 ± 0.02. For the reaction, 100 µL of SWP-Liq working solution (1 mg/mL) was introduced into 3.0 mL of the prepared DPPH reagent. The reaction was allowed to proceed for 30 min at ambient temperature under light-protected conditions, after which the residual DPPH absorbance was recorded at 517 nm. A control was processed through the same analytical sequence, except that the 100-µL SWP-Liq aliquot was substituted with an equal volume of distilled water. Radical-quenching efficiency was calculated according to Equation (2):

(2) $$\mathrm{Inhibition}\;(\%)=\left(\frac{A_{0}-A_{1}}{A_{0}}\right)\times 100$$

where *A*_0_ denotes the optical density of the control reaction, and *A*_1_ represents the corresponding value measured in the presence of SWP-Liq. To convert the DPPH response into antioxidant-equivalent values, three reference antioxidants were independently employed: Trolox (T), butylated hydroxyanisole (BHA), and α-tocopherol (α-TOC). Their respective calibration relationships were T: y = −0.0030x + 0.9565 (R^2^ = 0.9993), BHA: y = −0.0031x + 0.9424 (R^2^ = 0.9987), and α-TOC: y = −0.0011x + 0.9522 (R^2^ = 0.9983). The antioxidant capacity corresponding to each standard was obtained separately from these equations and reported as mg TE/L, mg BHAE/L, and mg α-TOCE/L, respectively.

#### 2.7.2. Determination of ABTS•^+^ Radical-Scavenging Activity

The ABTS•^+^-quenching potential of SWP-Liq was evaluated through an adaptation of the spectrophotometric assay reported by Ahmed et al. [30]. The radical cation was generated by reacting ABTS (7 mM) with potassium persulfate (2.45 mM) for 16–18 h under light-protected conditions. Immediately before analysis, the resulting radical solution was adjusted with methanol to give an optical density of 0.700 ± 0.02 at 734 nm. For each determination, 150 µL of SWP-Liq working solution (1 mg/mL) was introduced into 2850 µL of the adjusted ABTS•^+^ reagent. After allowing the reaction to proceed for 10 min at ambient temperature, the remaining ABTS•^+^ signal was monitored spectrophotometrically at 734 nm using a Multiskan SkyHigh microplate spectrophotometer (Thermo Fisher Scientific, Waltham, MA, USA). A parallel control was prepared through the same procedure, with 150 µL distilled water replacing the SWP-Liq aliquot.

Three reference antioxidants were independently used to convert the measured response into equivalent antioxidant capacities. The calibration relationships were y = −0.0026x + 0.6337 (R^2^ = 0.9988) for T, y = −0.0047x + 0.6276 (R^2^ = 0.9985) for BHA, and y = −0.0021x + 0.6285 (R^2^ = 0.9986) for α-TOC. Values calculated from the individual calibration models were expressed as mg TE/L, mg BHAE/L, and mg α-TOCE/L, respectively. The proportion of ABTS•^+^ neutralized by SWP-Liq was additionally calculated using the inhibition relationship provided in Equation (2).

#### 2.7.3. Determination of Fe^3+^ Reducing Power

The electron-donating ability of SWP-Liq toward the Fe^3+^/Fe^2+^ redox system was assessed by adapting the ferricyanide reduction assay originally introduced by Oyaizu [31]. The initial reaction medium constituted 500 µL SWP-Liq working solution (1 mg/mL), 500 µL phosphate buffer (0.2 M, pH 6.6), and 500 µL potassium ferricyanide [K_3_Fe(CN)_6_; 1%, *w*/*v*]. Reduction was promoted by holding this mixture at 50 °C for 20 min. Thereafter, 500 µL trichloroacetic acid (TCA; 10%, *w*/*v*) was introduced to terminate the reaction, and the resulting preparation was centrifuged at 3000 rpm for 10 min. From the clarified upper phase, a 500-µL portion was recovered and reacted with an equal volume of FeCl_3_ solution (0.1%, *w*/*v*). Color formation was allowed to develop for 10 min under ambient conditions, followed by spectrophotometric measurement at 700 nm using a Multiskan SkyHigh microplate spectrophotometer (Thermo Fisher Scientific, Waltham, MA, USA). Background correction was achieved with a reagent blank processed identically, except that deionized water was used in place of SWP-Liq. Quantification of the Fe^3+^-reducing response was independently referenced to T and BHA. The corresponding calibration models were y = 0.0086x + 0.0283 (R^2^ = 0.9976) for Trolox and y = 0.0328x + 0.0795 (R^2^ = 0.9964) for BHA. Activities calculated from these individual calibration relationships were reported as mg T equivalents per liter (mg TE/L) and mg BHA equivalents per liter (mg BHAE/L), respectively.

#### 2.7.4. Determination of Cu^2+^ Reducing Capacity

The capacity of SWP-Liq to reduce Cu^2+^ was evaluated by adapting the CUPRAC assay originally established by Apak et al. [32]. The assay medium was prepared by introducing 0.5 mL of SWP-Liq working solution (1 mg/mL) into a system containing 1.0 mL CuCl_2_ (10 mM), 1.0 mL ethanolic neocuproine (7.5 mM), and 1.0 mL ammonium acetate buffer adjusted to pH 7.0. Deionized water was subsequently used to bring the total reaction volume to 4.0 mL. Following vortex homogenization, color development proceeded for 30 min at ambient temperature under protection from light. Formation of the Cu^+^–neocuproine chromophore, reflecting reduction of Cu^2+^ by antioxidant constituents of SWP-Liq, was subsequently evaluated from its optical response at 450 nm using a Multiskan SkyHigh microplate spectrophotometer (Thermo Fisher Scientific, Waltham, MA, USA). Background absorbance was established through an otherwise identical reaction in which the 0.5-mL sample aliquot was substituted with deionized water. The reducing response was quantified independently against T and BHA. Their respective calibration functions were y = 0.0008x + 1.3067 (R^2^ = 0.9941) and y = 0.0088x + 1.3112 (R^2^ = 0.9971). Values derived from the corresponding calibration models were expressed as milligrams of T equivalents per liter (mg TE/L) and milligrams of BHA equivalents per liter (mg BHAE/L), respectively.

### 2.8. Determination of Anti-Inflammatory Activity

The ability of SWP-Liq to suppress 5-lipoxygenase (5-LOX) activity was assessed in a UV-compatible 96-well quartz microplate following the experimental approach of Yıldırım et al. [33]. The assay system in each well was assembled with 10 µL of SWP-Liq at the required test concentration, 20 µL ethanol, 20 µL borate buffer (pH 9.0), and 25 µL of 5-LOX solution (20,000 U/mL, Lipoxidase from Glycine max, Type V, L6632, Sigma-Aldrich, St. Louis, MO, USA) prepared in the same buffer. Before substrate addition, the enzyme–sample system was equilibrated at 25 °C for 5 min. Enzymatic conversion was subsequently triggered by introducing 100 µL linoleic acid (0.6 mM). Reaction progress was followed kinetically for 6 min through changes in optical density at 234 nm using a Multiskan SkyHigh microplate spectrophotometer (Thermo Fisher Scientific, Waltham, MA, USA). Indomethacin (INDO) was analyzed in parallel as the reference 5-LOX inhibitor. The extent of enzyme suppression was calculated as percentage inhibition according to Equation (2). Concentration-dependent inhibition data were then used to construct the response curve, from which the IC_50_ was estimated as the SWP-Liq concentration required to reduce 5-LOX activity by 50%.

### 2.9. Determination of Antidiabetic Activity

The potential of SWP-Liq to interfere with α-glucosidase activity was investigated using the enzyme-based assay of Ramakrishna et al. [34]. For each SWP-Liq concentration, an initial assay volume of 100 µL was assembled from 10 µL aqueous SWP-Liq, 40 µL sodium phosphate buffer (0.1 M, pH 6.9), and 50 µL of *Saccharomyces cerevisiae* α-glucosidase solution (1 U/mL; Type I, G5003, Sigma-Aldrich, St. Louis, MO, USA) with the enzyme previously diluted in the same buffer. The sample–enzyme system was allowed to equilibrate for 15 min at 25 °C. Enzymatic hydrolysis was then initiated by introducing 50 µL of *p*-nitrophenyl-α-D-glucopyranoside (pNPG; 5 mM), also prepared in sodium phosphate buffer, followed by a further 15-min reaction period at 25 °C. Optical density at 405 nm using a Multiskan SkyHigh microplate spectrophotometer (Thermo Fisher Scientific, Waltham, MA, USA). was measured with a microplate reader both before substrate conversion and at the end of the reaction period. Acarbose was assayed under comparable conditions as the reference α-glucosidase inhibitor. Enzyme suppression was expressed as percentage inhibition according to Equation (2), taking into account the absorbance changes measured for the control and SWP-Liq-treated systems. The resulting concentration–response relationship was subsequently used to estimate IC_50_, corresponding to the SWP-Liq concentration associated with a 50% decrease in α-glucosidase activity.

### 2.10. Determination of Antibacterial Activity by the Agar Well Diffusion Method

The growth-suppressing effect of SWP-Liq was examined against *E. coli*, *P. aeruginosa*, *E. faecalis*, and *S. aureus* by adapting the agar well diffusion protocol reported by Kobya et al. [35]. To prepare the bacterial inoculum, each strain was first propagated in nutrient broth (NB) for 16–18 h, thereby providing fresh, metabolically active cultures for testing. Aliquots of these cultures were distributed across nutrient agar (NA) surfaces, and the inoculated plates were left undisturbed for a short period to facilitate even establishment of the bacterial lawn. Wells measuring 6 mm in diameter were subsequently formed in the agar, and each was supplemented with 50 µL of SWP-Liq prepared at either 8 or 4 mg/mL. The inoculated plates remained at 25 °C for 1 h before incubation, providing sufficient time for the test material to diffuse radially into the surrounding agar matrix. Bacterial growth was then allowed to proceed at 37 °C for 18 h. After incubation, antibacterial response was quantified by determining the diameter of the clear growth-inhibition area surrounding each well with a digital caliper. Ampicillin at 20 µg/mL was included in parallel as the reference antibacterial compound.

### 2.11. Statistical Analyses

All experiments were performed using a single homogenized SWP-Liq preparation obtained from one pyrolysis batch. For each analytical assay, measurements were independently repeated three times at the technical level (*n* = 3) to assess analytical repeatability. The reported replicates represent repeated measurements of the same homogenized preparation. Experimental values are presented as arithmetic means ± standard deviations (SD), with the SD reflecting variability among technical measurements. For comparisons between two groups, Student’s *t*-test was used, whereas comparisons involving three or more groups were performed using one-way analysis of variance (ANOVA) followed by Tukey’s honestly significant difference (HSD) post hoc test. Statistical significance was set at *p* < 0.05. For enzyme-inhibition assays, concentration–response data were analyzed by nonlinear regression using GraphPad Prism 10 (GraphPad Software, Boston, MA, USA), and IC_50_ values were estimated from the fitted concentration–response curves. Because the present study was based on a single pyrolysis-derived preparation, statistical findings are interpreted as assay-level differences within the investigated preparation and not as evidence of biological or between-batch variability.

## 3. Results and Discussion

### 3.1. Evaluation of TPC, TFC, and TTC

In this study, the TPC, TFC, and TTC of the pyrolysis-derived liquid fraction obtained from SWP-Liq were evaluated, and the results are presented in Table 1. The findings demonstrated that SWP-Liq exhibited measurable phenolic-, flavonoid-, and triterpenoid-equivalent responses, indicating that this agro-industrial by-product can be converted into a chemically evaluable liquid fraction [36].

The TPC and TFC of SWP-Liq were determined as 218.47 ± 22.40 mg GAE/g and 79.13 ± 3.55 mg QE/g, respectively, demonstrating measurable phenolic- and flavonoid-equivalent responses in the pyrolysis-derived fraction. Previous studies have reported associations between phenolic and flavonoid constituents and antioxidant responses in plant- and silkworm-derived materials [14,22,37]. From a mechanistic perspective, phenolic compounds can participate in redox reactions through electron- and hydrogen-donating processes, while the stabilization of the resulting phenoxyl radicals by resonance contributes to their radical-scavenging behavior. These chemical properties provide a plausible basis for the contribution of phenolic constituents to the antioxidant responses subsequently observed for SWP-Liq. Because the Folin–Ciocalteu and TFC assays provide equivalent colorimetric responses rather than molecular identification, these compositional findings are best considered together with the LC–MS/MS profile, which confirmed the presence of several phenolic constituents in SWP-Liq.

Taken together, the TPC and TFC results demonstrate that SWP-Liq exhibits measurable Folin–Ciocalteu and flavonoid-equivalent colorimetric responses, suggesting the presence of redox-active constituents in the liquid fraction. Nevertheless, these assay-based equivalent values alone do not establish SWP-Liq as a natural antioxidant source or demonstrate its suitability for nutraceutical, cosmetic, or functional product applications. Instead, the findings provide a basis for further investigation of this waste-derived fraction within the framework of sustainable biomass valorization [38]. Accordingly, SWP-Liq may be regarded as a thermochemically derived intermediate fraction that warrants further chemical, biological, and safety evaluation before any specific application can be proposed.

The TTC of SWP-Liq was determined as 55.54 ± 6.15 mg EE/g, demonstrating a measurable triterpenoid-equivalent response in the pyrolysis-derived liquid fraction. Triterpenoids constitute an important class of secondary metabolites with reported antioxidant and anti-inflammatory properties, including modulation of NF-κB signaling and inflammatory mediators such as COX-2, iNOS, and pro-inflammatory cytokines [39,40,41,42,43]. However, the interpretation of the present TTC response should also consider the thermochemical nature of SWP-Liq. Pyrolysis can induce degradation, rearrangement, and transformation of structurally complex phytochemicals, and the thermal stability of pentacyclic triterpenes varies according to molecular structure and thermal exposure [44,45]. Because silkworm feeding waste contains mulberry leaf-derived residues, plant-derived constituents may contribute to the starting biomass and its thermochemical products [9,11,46]. Thus, the observed TTC response may originate from retained triterpenoid-related structures and/or thermochemical transformation products capable of reacting in the vanillin–perchloric acid assay. Comparative profiling before and after pyrolysis would be required to distinguish between these possibilities.

Collectively, the TPC, TFC, and TTC results indicate that SWP-Liq is a chemically complex liquid fraction exhibiting phenolic-, flavonoid-, and triterpenoid-equivalent responses. Together with the targeted LC–MS/MS findings, these compositional characteristics provide a chemical basis for the biological responses evaluated in the subsequent assays. From a waste-valorization perspective, the recovery of a chemically active fraction from silkworm feeding waste supports further investigation of pyrolysis as an approach for converting this low-value residue into a potentially useful bio-based material.

### 3.2. LC–MS/MS-Based Chemical Composition Analysis

**Figure 2:**
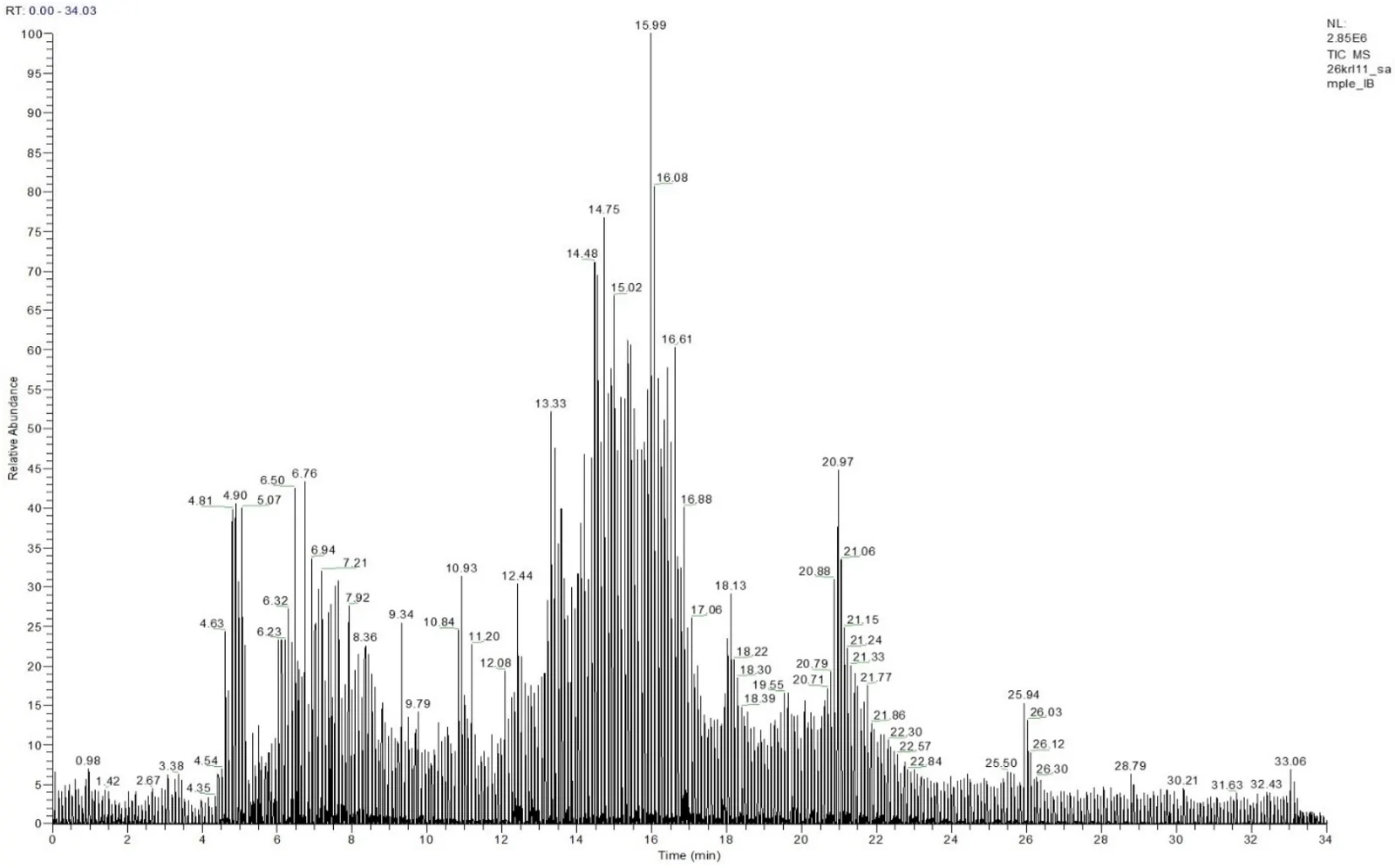
Representative total ion current (TIC) chromatogram showing the overall chemical profile of the SWP-Liq.

In this study, the targeted chemical profile of SWP-Liq was characterized by LC–MS/MS analysis. Of the 35 reference compounds included in the analytical panel, only five target analytes met the criteria for quantitative determination, whereas the remaining compounds were not quantified because their analytical responses were below the applicable detection (LOD) and/or quantification (LOQ) thresholds. The quantitative results for the phenolic compounds detected in SWP-Liq are presented in Table 2, while a representative chromatographic profile obtained from the LC–MS/MS analysis is shown in Figure 2.

The targeted LC–MS/MS analysis revealed that the quantified analytes in SWP-Liq consisted predominantly of phenolic acids and their derivatives. Among the quantified target analytes, *p*-coumaric acid showed the highest concentration (1599.71 µg/g), followed by chlorogenic acid (871.43 µg/g), ferulic acid (285.50 µg/g), salicylic acid (39.72 µg/g), and luteolin-7-glucoside (0.50 µg/g). *p*-Coumaric and ferulic acids are hydroxycinnamic acid derivatives, chlorogenic acid is a caffeoylquinic acid ester, salicylic acid is a hydroxybenzoic acid derivative, and luteolin-7-glucoside belongs to the flavone glycoside subclass. The limited detection of the other targeted compounds may reflect their absence or low abundance, thermal transformation during pyrolysis, or limitations associated with the analytical scope and matrix. These possibilities cannot be distinguished on the basis of the present targeted analysis alone. Pyroligneous acid and related pyrolysis-derived liquids have been reported to exhibit highly variable chemical compositions depending on the feedstock and pyrolysis conditions and to contain complex mixtures of phenols, organic acids, furans, and other oxygenated organic compounds [6]. Accordingly, the phenolic acids quantified in SWP-Liq may represent compounds retained from the original biomass, products of thermal degradation, or compounds formed through secondary thermochemical reactions during pyrolysis; distinguishing among these possible origins would require comparative profiling of the pre- and post-pyrolysis materials.

The relatively high level of *p*-coumaric acid among the quantified target analytes is noteworthy. *p*-Coumaric acid is a hydroxycinnamic acid for which antioxidant and antimicrobial properties have been reported in various experimental systems [47]. Chlorogenic acid, which represented the second most abundant quantified target analyte, has also been widely associated with radical-scavenging and redox-related activities [48]. Similarly, ferulic acid is a hydroxycinnamic acid capable of participating in electron- and hydrogen-transfer reactions and has been associated with antioxidant and antimicrobial properties [49]. Recent studies have further highlighted the biological activities associated with phenolic acids such as chlorogenic, ferulic, and *p*-coumaric acids [50].

The targeted phenolic profile can be considered together with the antioxidant responses obtained for SWP-Liq. The DPPH and ABTS•^+^ radical-scavenging responses and Fe^3+^- and Cu^2+^-reducing capacities are chemically consistent with the presence of redox-active constituents, including the phenolic acids detected by LC–MS/MS. Polyphenols can participate in redox processes through hydrogen-atom donation and electron-transfer reactions, while resonance stabilization of the resulting phenoxyl radicals can reduce their propensity to propagate radical reactions [51,52]. Accordingly, the hydroxycinnamic acids identified in SWP-Liq, particularly *p*-coumaric, chlorogenic, and ferulic acids, provide a chemically plausible contribution to the observed radical-scavenging and reducing responses. Nevertheless, because compound-specific activity assays and correlation analyses were not performed, the magnitude of their individual contributions cannot be determined. Interactions among hydroxycinnamic acids have been reported to influence the overall response of complex antioxidant systems [53], suggesting that the antioxidant activity of SWP-Liq may reflect the combined redox behavior of multiple constituents rather than the effect of a single dominant compound.

The antibacterial responses of SWP-Liq may also be considered in relation to its targeted phenolic profile. Chlorogenic, *p*-coumaric, ferulic, and salicylic acids detected in the sample have previously been associated with antibacterial activity. Phenolic acids have been reported to affect bacterial membrane integrity, permeability, and intracellular homeostasis through several mechanisms [54,55]. In particular, hydroxycinnamic acids such as ferulic and *p*-coumaric acids have been shown to inhibit bacterial growth and affect membrane integrity in experimental systems [55,56]. Chlorogenic acid has likewise been associated with several biological activities, including antibacterial effects [57]. These membrane-related effects provide a plausible mechanistic context for the antibacterial response observed for SWP-Liq. In addition to the detected phenolic acids, other oxygenated organic constituents generated during pyrolysis may contribute to the antibacterial response, suggesting that the observed activity may reflect the combined effects of multiple constituents within the chemically complex pyrolysis-derived fraction [50].

Another notable feature of Table 2 is that most flavonoids included in the targeted analytical panel were not quantifiable, whereas luteolin-7-glucoside was detected at only 0.50 µg/g. The targeted LC–MS/MS analysis in the present study was specifically intended to characterize the phenolic constituents present in the final SWP-Liq product, since the primary objective was to evaluate the chemical and biological properties of the pyrolysis-derived material. Pyrolysis involves thermal degradation, fragmentation, and secondary reactions that can substantially modify the chemical composition of the original biomass [58]. Accordingly, the pre-pyrolysis feedstock was not subjected to LC–MS/MS profiling because the study was not designed as a comparative pre- versus post-pyrolysis investigation; likewise, the biological activity of the untreated feedstock was not evaluated. One possible explanation for the limited quantification of flavonoids is their thermal susceptibility under pyrolysis conditions; however, this cannot be established from the present data. Similarly, the predominance of phenolic acids among the quantified target analytes may reflect differences in their initial abundance, thermal behavior, partitioning into the condensed liquid fraction, or analytical detectability. Phenolic acids such as chlorogenic, ferulic, and *p*-coumaric acids have also been reported in other thermochemically or biologically derived acidic liquid matrices [59]. Therefore, the quantification of only five of the 35 targeted compounds should not be interpreted as evidence that the remaining compounds were absent from the original feedstock, as they may have been thermally transformed or may have been present below the analytical detection or quantification limits in the final product. Overall, the targeted LC–MS/MS results indicate a profile dominated by hydroxycinnamic acids among the quantified target analytes rather than demonstrating enrichment of these compounds during pyrolysis. Given the chemical complexity of pyrolysis-derived liquids, compounds below the analytical thresholds and constituents outside the targeted LC–MS/MS panel may also contribute to the overall chemical and biological properties of SWP-Liq.

### 3.3. Evaluation of DPPH and ABTS•^+^ Radical-Scavenging Activities

**Figure 3:**
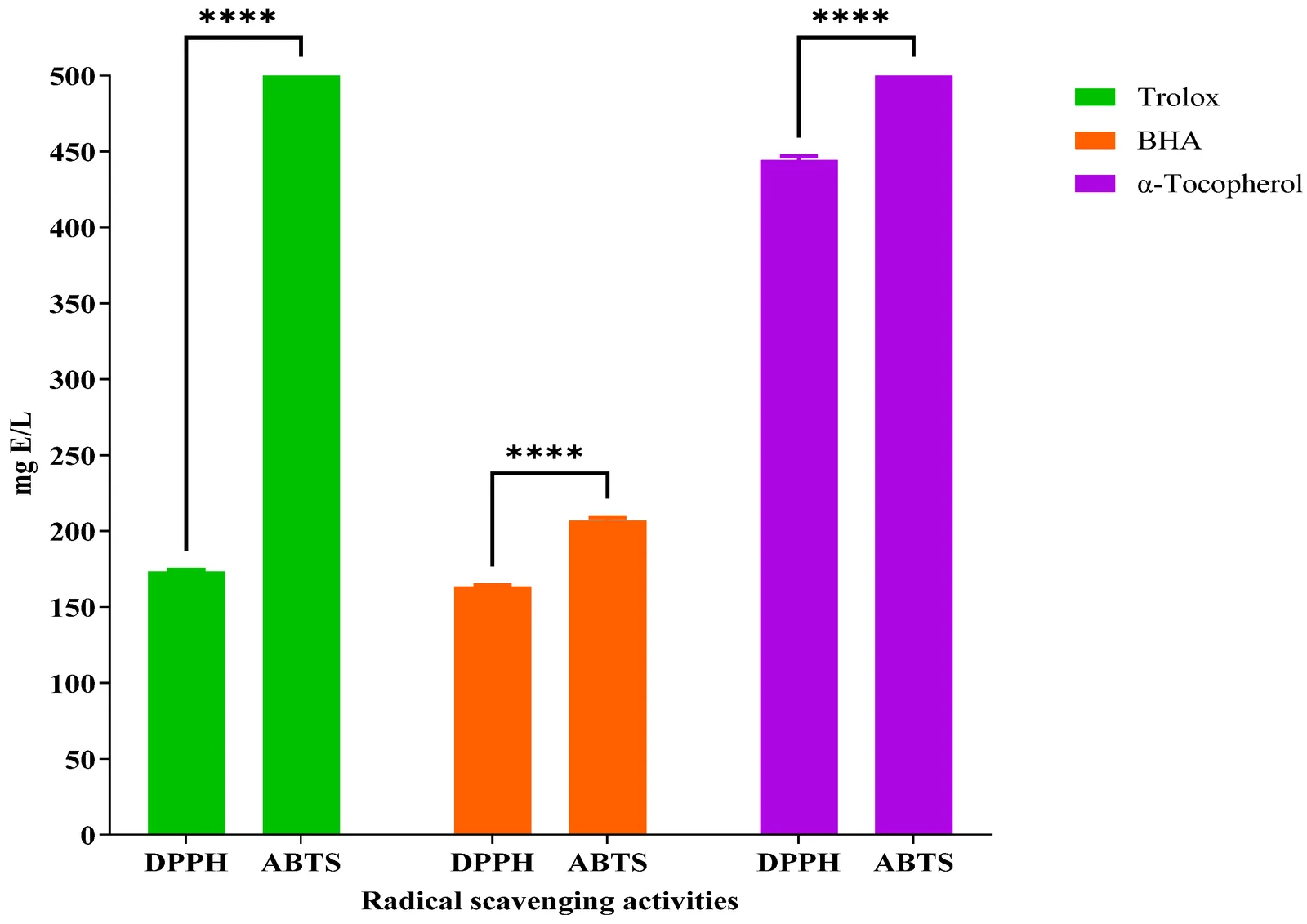
Antioxidant capacity of SWP-Liq determined by DPPH and ABTS•^+^ assays and expressed as Trolox, BHA, and α-TOC equivalents. Data are reported as mean ± SD of three technical replicate measurements (*n* = 3). Significant differences between DPPH and ABTS•^+^ antioxidant capacities within each calibration standard are indicated by **** *p* < 0.0001.

The antioxidant response of SWP-Liq was evaluated using the complementary DPPH and ABTS•^+^ radical-scavenging assays, and the results are presented in Figure 3. Because these assays involve different radical systems and reaction conditions, they provide complementary measures of radical-scavenging behavior rather than directly interchangeable measures of antioxidant activity. SWP-Liq exhibited a DPPH radical-scavenging activity of 42.06 ± 0.31%, demonstrating a measurable radical-scavenging response under the applied assay conditions. When expressed relative to the respective reference antioxidant calibration curves, the DPPH response corresponded to 173.39 ± 1.97 mg TE/L, 163.56 ± 1.94 mg BHAE/L, and 444.33 ± 2.50 mg α-TOCE/L. These values represent alternative expressions of the same radical-scavenging response relative to different reference standards and therefore should not be interpreted as directly comparable antioxidant capacity values.

In the ABTS•^+^ assay, SWP-Liq exhibited 92.43 ± 1.04% radical scavenging, representing a numerically higher percentage response than that observed in the DPPH assay under the respective experimental conditions. However, because the two assays differ in radical chemistry and reaction conditions, these percentages should not be interpreted as directly comparable measures of intrinsic antioxidant potency. The antioxidant capacity determined using the ABTS•^+^ assay corresponded to 674.92 ± 6.27 mg TE/L, 206.73 ± 2.38 mg BHAE/L, and 790.68 ± 9.08 mg α-TOCE/L. The pronounced difference between the DPPH (42.06%) and ABTS•^+^ (92.43%) responses indicates that the radical-scavenging behavior of SWP-Liq is strongly dependent on the assay system. This discrepancy may reflect differences in radical chemistry, solvent environment, accessibility of redox-active constituents, analyte compatibility, and reaction kinetics [60,61]. In particular, ABTS•^+^ can respond to a broader range of both hydrophilic and lipophilic antioxidant constituents, whereas DPPH is generally applied in an organic-solvent environment. For a chemically heterogeneous pyrolysis-derived fraction such as SWP-Liq, these differences may substantially influence the accessibility and reactivity of individual constituents toward the respective radicals. Therefore, the markedly higher ABTS•^+^ response should not be interpreted as evidence of uniformly high antioxidant potency, but rather as a stronger radical-scavenging response under the specific conditions of the ABTS•^+^ assay. The magnitude of the discrepancy also demonstrates the importance of using complementary antioxidant assays when evaluating complex pyrolysis-derived matrices rather than relying on a single radical-scavenging system.

The observed radical-scavenging responses may be associated, at least in part, with the phenolic constituents detected in SWP-Liq. LC–MS/MS analysis identified chlorogenic, *p*-coumaric, ferulic, and salicylic acids, all of which possess chemical functionalities capable of participating in redox reactions. In particular, chlorogenic acid and the hydroxycinnamic acids *p*-coumaric and ferulic acids have been associated with antioxidant properties, including radical scavenging and inhibition of oxidative processes [48]. Similarly, pyroligneous liquids have been reported to contain phenolic and other oxygenated organic constituents that can contribute to measurable antioxidant responses [62]. The hydroxylated aromatic structures of these phenolic acids can facilitate hydrogen-atom and electron-transfer reactions, while resonance stabilization of the resulting phenoxyl radicals provides a plausible chemical basis for their contribution to the radical-scavenging behavior of SWP-Liq. In a chemically complex pyrolysis-derived fraction, the overall antioxidant response may reflect the combined redox behavior of these phenolic acids together with other oxygenated constituents rather than the activity of a single compound. Overall, these findings indicate that SWP-Liq contains constituents capable of participating in different radical-scavenging systems and support further characterization of silkworm feeding waste-derived pyrolysis liquids as chemically complex fractions with in vitro antioxidant potential.

### 3.4. Evaluation of Fe^3+^- and Cu^2+^-Reducing Capacities

**Figure 4:**
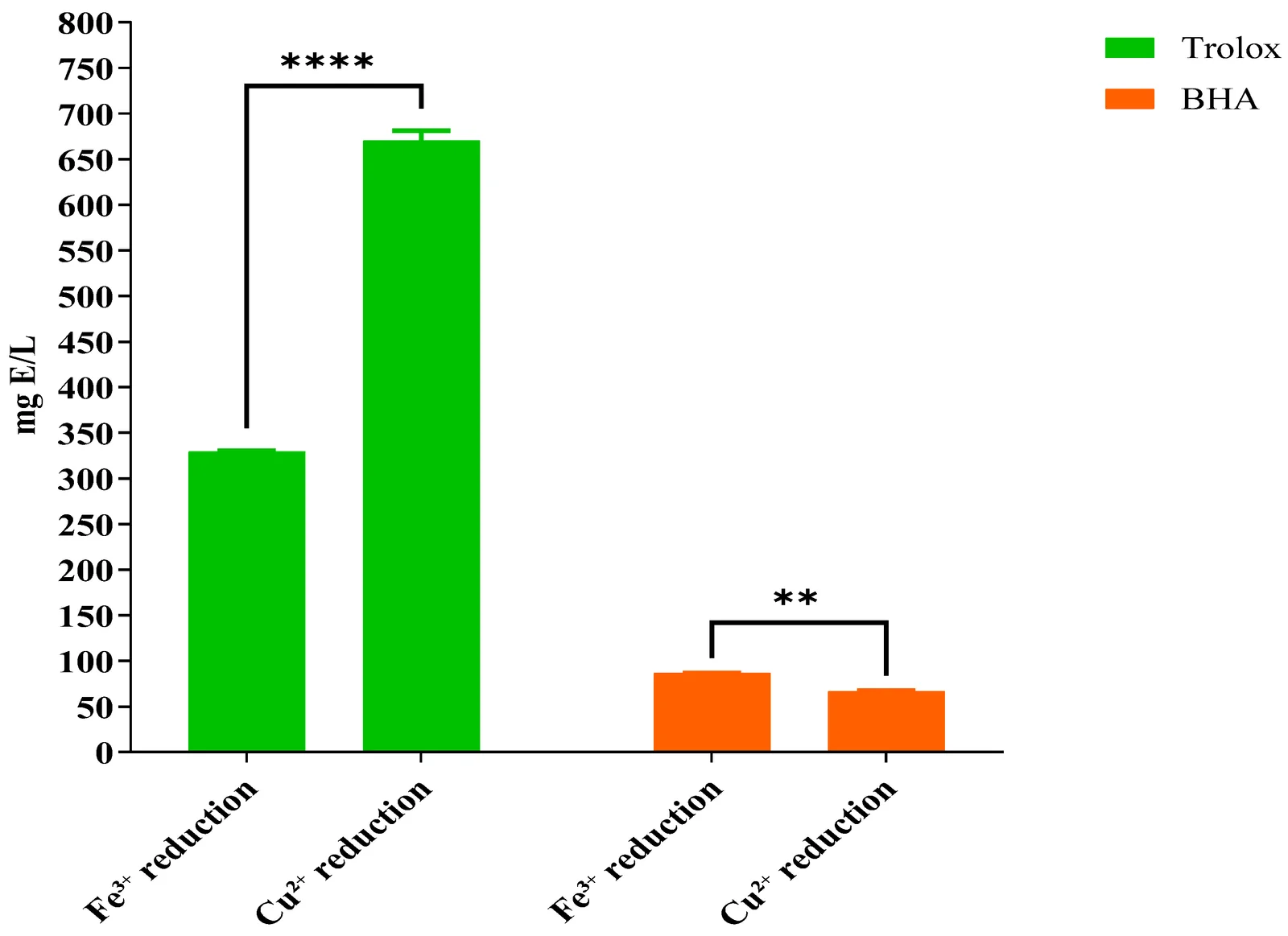
Fe^3+^- and Cu^2+^-reducing capacities of SWP-Liq determined using T and BHA calibration curves. Results are expressed as mg TE/L and mg BHAE/L (mean ± SD, *n* = 3). Significant differences between Fe^3+^- and Cu^2+^-reducing capacities within each calibration standard are indicated by ** *p* < 0.01 and **** *p* < 0.0001.

The electron-transfer-based antioxidant response of SWP-Liq was evaluated using Fe^3+^- and Cu^2+^-reducing assays, and the results are presented in Figure 4. SWP-Liq exhibited measurable reducing capacity in both assay systems. In the Fe^3+^-reducing assay, the antioxidant capacity was determined as 329.35 ± 1.17 mg TE/L and 86.58 ± 0.31 mg BHAE/L. This response indicates that SWP-Liq contains constituents capable of participating in electron-transfer reactions under the applied ferricyanide-reducing assay conditions [63].

In the Cu^2+^-reducing assay, the antioxidant capacity of SWP-Liq was calculated as 670.33 ± 11.11 mg TE/L and 66.53 ± 1.11 mg BHAE/L. The difference between the Fe^3+^- and Cu^2+^-reducing responses was statistically significant when expressed as Trolox equivalents (*p* < 0.0001) and BHA equivalents (*p* < 0.01). The different response patterns observed in the Fe^3+^- and Cu^2+^-reducing assays are consistent with their distinct reaction chemistries and may reflect differences in redox potential, reaction conditions, reagent systems, and the physicochemical characteristics of the responding constituents [64]. These assay-dependent differences suggest that the chemically diverse redox-active constituents of SWP-Liq interact differently with the two metal ion-based systems.

These findings can also be considered in relation to the targeted phenolic profile determined by LC–MS/MS. Chlorogenic, *p*-coumaric, ferulic, and salicylic acids detected in SWP-Liq belong to classes of phenolic compounds that have been associated with antioxidant and reducing properties [48]. In particular, chlorogenic acid has been associated with redox activity, while hydroxycinnamic acid derivatives such as ferulic and *p*-coumaric acids can participate in electron- and/or hydrogen-transfer reactions [65]. More broadly, the antioxidant behavior of polyphenols can involve not only radical-scavenging reactions but also electron-transfer and metal ion-related redox processes [52]. Accordingly, the phenolic acids identified by LC–MS/MS provide a plausible chemical basis for at least part of the Fe^3+^- and Cu^2+^-reducing responses, while the overall reducing behavior of SWP-Liq is likely to reflect the collective contribution of multiple redox-active constituents within the pyrolysis-derived fraction. Overall, these results demonstrate the presence of redox-active constituents in the pyrolysis-derived liquid fraction and support further evaluation of SWP-Liq as a chemically and functionally characterizable bio-based product of silkworm feeding waste valorization.

### 3.5. Evaluation of the Anti-Inflammatory Potential of SWP-Liq

In the present study, the anti-inflammatory potential of SWP-Liq was evaluated based on 5-LOX inhibition. The results were interpreted on the basis of IC_50_ values (µg/mL) and compared with INDO as the positive control (Table 3). As shown in Table 3, SWP-Liq exhibited a measurable 5-LOX inhibitory response, with an IC_50_ value of 33.05 ± 1.10 µg/mL. The higher IC_50_ value of SWP-Liq compared with that of indomethacin indicates a lower inhibitory potency. 5-LOX catalyzes key steps in the conversion of arachidonic acid to leukotrienes, which are important lipid mediators involved in inflammatory processes. Therefore, the observed inhibition provides a mechanistically relevant enzyme-based indication of the anti-inflammatory potential of SWP-Liq and supports further evaluation of this response in cellular and in vivo models.

Anti-inflammatory effects have previously been reported for several silkworm-derived fractions. Silkworm larval by-product powder (SLPY) has been shown to reduce the expression of TNF-α, IL-1β, IL-8, and COX-2 and to modulate NF-κB/MAPK signaling in cellular and in vivo experimental models [8]. Similarly, silkworm pupa protein-derived peptides (SPP) have been reported to attenuate inflammatory responses through modulation of NF-κB, MAPK, and PI3K signaling pathways [66], while anti-inflammatory responses have also been reported for silkworm hemocyte/hemolymph-derived preparations [67]. Silk-derived materials, including fibroin- and sericin-based preparations, have also been investigated in relation to inflammatory responses and wound healing [68,69]. Collectively, these studies demonstrate that different silkworm-derived materials can exhibit anti-inflammatory responses in a variety of experimental systems.

The 5-LOX inhibitory response observed for SWP-Liq provides an additional enzyme-based endpoint to the biological activities previously reported for silkworm-derived materials. The thermochemical nature of SWP-Liq should also be considered when interpreting this response, because pyrolysis can generate a chemically heterogeneous mixture containing retained, transformed, and newly formed constituents. The phenolic acids identified by LC–MS/MS, together with other uncharacterized components of the pyrolysis-derived fraction, may collectively contribute to the observed 5-LOX inhibition. Identification of the constituents primarily responsible for this activity will require compound-directed or fractionation-based investigations.

### 3.6. Evaluation of the Antidiabetic Potential of SWP-Liq

In the present study, the antidiabetic potential of the pyrolysis-derived liquid fraction obtained from silkworm feeding waste (SWP-Liq) was evaluated based on α-glucosidase inhibition. α-Glucosidase catalyzes the terminal hydrolysis of dietary oligosaccharides and disaccharides to absorbable monosaccharides; therefore, inhibition of this enzyme can delay intestinal carbohydrate digestion and attenuate postprandial glucose release [70]. The results were interpreted according to IC_50_ values (µg/mL) and compared with acarbose as the positive control (Table 4). As shown in Table 4, SWP-Liq exhibited α-glucosidase inhibitory activity with an IC_50_ value of 1682.3 ± 3.2 µg/mL, whereas acarbose showed a substantially lower IC_50_ value of 272.0 ± 1.5 µg/mL. These results demonstrate that SWP-Liq has considerably weaker α-glucosidase inhibitory potency than acarbose. Despite its lower potency relative to acarbose, the observed IC_50_ demonstrates that SWP-Liq possesses measurable α-glucosidase inhibitory activity under the experimental conditions employed. Thus, the biological significance of this finding lies in the presence of an enzyme-inhibitory response in the pyrolysis-derived fraction, while its relatively high IC_50_ should be considered when evaluating the strength of this activity.

Previous studies have reported α-glucosidase inhibitory and glucose-related biological responses for several silkworm-derived materials. Wang et al. demonstrated that ethanolic extracts of non-sericin components from silkworm cocoon shells inhibited α-glucosidase, with IC_50_ values as low as 110 µg/mL for some cocoon types [71]. Flavonoid-rich ethanolic extracts from green silkworm cocoons have also been shown to inhibit α-glucosidase and α-amylase in vitro [37], while improvements in glucose-related metabolic parameters have been reported in diabetic animal models treated with green cocoon extracts [72]. These findings demonstrate that different sericulture-derived fractions can exhibit glucose metabolism-related biological activities, although their mechanisms and chemical compositions differ substantially [15,72].

Compared with these previously studied silkworm-derived extracts, SWP-Liq exhibited weaker α-glucosidase inhibition. However, direct comparison of IC_50_ values among studies should be approached cautiously because differences in sample preparation, enzyme source, substrate concentration, and assay conditions can substantially influence the measured inhibitory response. Moreover, SWP-Liq differs fundamentally from conventional solvent-derived, flavonoid-rich extracts because it represents a chemically complex fraction generated through thermochemical conversion. This distinction may contribute to differences in both chemical composition and α-glucosidase inhibitory potency relative to previously studied sericulture-derived extracts.

Other silkworm-derived materials have also been investigated for glucose-related metabolic effects. Silkworm powder, fibroin, and sericin have been reported to reduce fasting plasma glucose in diabetic rats [73], while sericulture-derived products have shown postprandial antihyperglycemic responses in experimental silkworm models [7]. Silkworm pupae have additionally been investigated for their nutritional, phenolic, antioxidant, and metabolic properties [14,74]. These findings provide a broader biological context for sericulture-derived materials; however, SWP-Liq differs chemically from these conventional preparations because pyrolysis can induce degradation, rearrangement, and formation of new thermochemical products from the original biomass [75]. Consequently, its α-glucosidase inhibitory response may arise from a chemically distinct mixture of retained and thermally transformed constituents.

The 5-LOX and α-glucosidase assays employed in the present study were cell-free enzyme-based assays and therefore characterize direct enzyme inhibition rather than cellular efficacy or safety. Cytotoxicity and selectivity were not evaluated in the present study, representing an important consideration for subsequent biological evaluation. Future studies combining relevant cell-based models with cytotoxicity and selectivity assessments will be necessary to determine whether the observed enzyme-inhibitory responses can be achieved at concentrations compatible with cell viability.

### 3.7. Evaluation of the Antibacterial Potential of SWP-Liq

**Figure 5:**
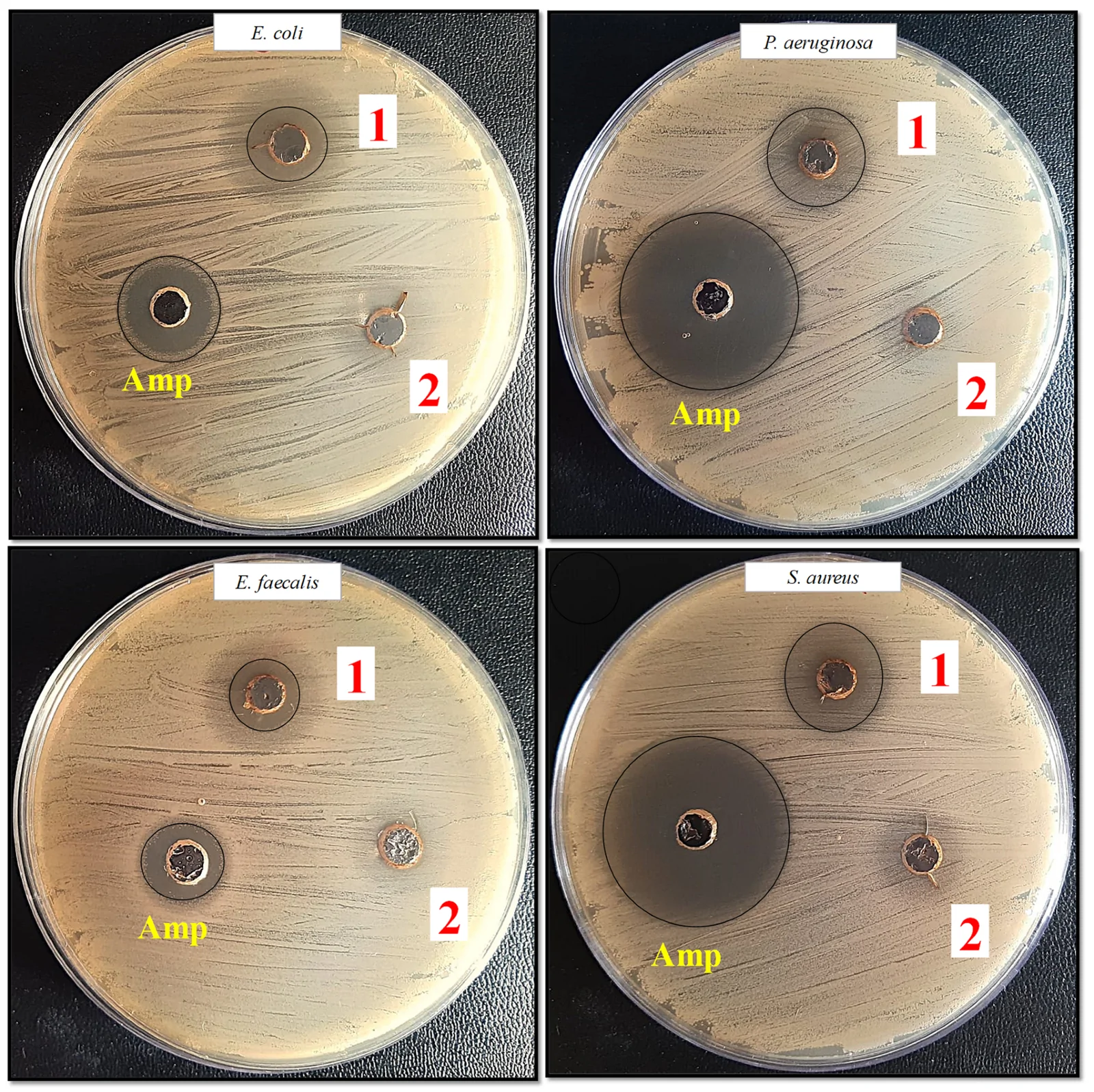
Representative images of Petri plates showing the antibacterial response of the tested samples (1: (SWP-Liq: 8 mg/mL), 2: (SWP-Liq: 4 mg/mL) ve Amp (Ampicillin): 20 µg/mL).

The antibacterial potential of SWP-Liq was evaluated against *E. coli*, *P. aeruginosa*, *S. aureus*, and *E. faecalis*. Based on preliminary experiments, the agar well diffusion assays were performed at SWP-Liq concentrations of 8 and 4 mg/mL, and the resulting inhibition zone diameters are presented in Table 5. Representative images of the agar plates obtained after incubation are shown in Figure 5. SWP-Liq produced measurable inhibition zones against all tested bacterial strains at 8 mg/mL, whereas no inhibition zones were observed at 4 mg/mL under the applied assay conditions.

At 8 mg/mL, SWP-Liq produced inhibition zones of 13.6 ± 0.9 mm against *E. coli*, 17.2 ± 1.1 mm against *P. aeruginosa*, 17.4 ± 1.0 mm against *S. aureus*, and 12.5 ± 0.8 mm against *E. faecalis*. The largest mean inhibition zones were observed for *S. aureus* and *P. aeruginosa*, whereas the smallest was observed for *E. faecalis*. These differences indicate species-dependent susceptibility to SWP-Liq under the applied agar diffusion conditions. Pyrolysis-derived liquids are chemically complex matrices that may contain phenolic compounds, organic acids, furans, ketones, and various oxygenated aromatic compounds, with their composition strongly dependent on the feedstock and pyrolysis conditions [5,18,20]. Accordingly, the antibacterial response of SWP-Liq may reflect the combined contribution of the phenolic constituents identified by targeted LC–MS/MS and other oxygenated components of the chemically complex pyrolysis-derived fraction. Özgen et al. investigated the antibacterial activity of pyrolysis liquids obtained from *Tenebrio molitor* L. adult- and larval-derived biowaste against two Gram-negative (*E. coli* and *P. aeruginosa*) and two Gram-positive (*E. faecalis* and *S. aureus*) strains using the agar well diffusion method. At the reported concentration of 20 mg/L, the pyrolysis liquid produced inhibition zones against all tested strains, with the largest zones observed against *S. aureus* (29.5 ± 0.3 mm) and *P. aeruginosa* (23.5 ± 0.2 mm) [24]. The present study likewise showed the largest mean inhibition zones against *S. aureus* and *P. aeruginosa*.

Gram-negative bacteria are known to exhibit lower permeability to many antimicrobial agents due to the lipopolysaccharide (LPS) layer present in their outer membrane and therefore generally display a more resistant cell envelope structure [76]. Despite this permeability barrier, SWP-Liq produced an inhibition zone of 17.2 ± 1.1 mm against *P. aeruginosa* at 8 mg/mL. Phenolic acids and other oxygenated constituents reported in pyrolysis-derived liquids may interact with bacterial cell envelopes and disturb membrane-associated processes, providing a plausible mechanistic context for the observed growth inhibition. With respect to Gram-positive bacteria, the inhibition zone observed particularly against *S. aureus* (17.4 ± 1.0 mm) indicates that SWP-Liq can also exert a measurable effect against this bacterial group. It has been reported that pyrolysis liquids and phenolic-containing natural products may produce more pronounced effects against Gram-positive bacteria, which may be associated with their relatively simpler cell wall organization and destabilization of the peptidoglycan layer [77]. In contrast, the lower inhibition observed against *E. faecalis* (12.5 ± 0.8 mm) may be related to the relatively more tolerant nature of this species.

As expected, ampicillin produced larger inhibition zones than SWP-Liq against all tested strains. Together with the absence of inhibition at 4 mg/mL, this comparison indicates that the antibacterial response of SWP-Liq was modest under the applied experimental conditions. Nevertheless, the growth inhibition observed at 8 mg/mL confirms the presence of antibacterial activity in the pyrolysis-derived fraction and supports further characterization of the constituents associated with this response.

The agar well diffusion assay used in the present study was intended as an initial screening approach for antibacterial activity. The inhibition zones therefore provide evidence of growth-inhibitory activity under the applied conditions, while quantitative determination of minimum inhibitory concentration (MIC) and minimum bactericidal concentration (MBC) in future studies would enable more precise characterization of the inhibitory and bactericidal potency of SWP-Liq.

## 4. Conclusions

This study demonstrated that silkworm feeding waste can be converted through pyrolysis into a liquid fraction amenable to chemical and biological evaluation. SWP-Liq exhibited phenolic-, flavonoid-, and triterpenoid-equivalent colorimetric responses together with a targeted LC–MS/MS phenolic profile and showed in vitro antioxidant, enzyme-inhibitory, and antibacterial activities. These findings indicate that low-value organic residues generated during silkworm rearing may be considered not only as waste requiring disposal but also as alternative feedstocks for pyrolysis-based valorization strategies.

To the best of our knowledge, this study represents one of the earliest integrated chemical and biological evaluations specifically of a pyrolysis-derived liquid fraction obtained from silkworm feeding waste, combining targeted phenolic profiling with antioxidant, 5-LOX- and α-glucosidase-inhibitory, and antibacterial assessments. Future studies should prioritize comparative chemical profiling of the pre-pyrolysis feedstock and the resulting SWP-Liq to distinguish retained constituents from thermal degradation products and compounds formed through secondary thermochemical reactions. Broader chemical profiling beyond the present targeted LC–MS/MS panel would further define the composition of the pyrolysis-derived fraction and facilitate activity-guided identification of constituents associated with the observed biological responses. For biological validation, MIC and MBC determinations should be performed to quantify antibacterial potency, while cell-based cytotoxicity and selectivity assessments are needed to establish the biological relevance of the enzyme-inhibitory responses. In parallel, independent pyrolysis batches should be investigated to establish process reproducibility. Subsequently, systematic optimization of key pyrolysis parameters, including temperature, heating rate, and residence time, using experimental design approaches such as response surface methodology (RSM), could be employed to evaluate their individual and interactive effects on product yield, chemical composition, and biological activity and to identify suitable processing conditions for the desired characteristics of SWP-Liq.

## Figures and Tables

**Table 1:** Quantitative assessment of TPC, TFC and TTC in the analyzed sample *.

Sample	TPC (mg GAE/g)	TFC (mg QE/g)	TTC (mg EE **/g)
SWP-Liq	218.47 ± 22.40	79.13 ± 3.55	55.54 ± 6.15

* TPC: Total Phenolic Content, TFC: Total Flavonoid Content, TTC: Total Triterpenoid Content. SWP-Liq: pyrolysis-derived liquid fraction obtained from silkworm feeding waste. The mean ± SD of three technical replicate measurements (*n* = 3) is used to express the data. ** E: Escin.

**Table 2:** LC–MS/MS quantification of targeted phenolic constituents in SWP-Liq.

No	Compounds	RT (min) ^a^	Precursor Ion (*m*/*z*)	Product Ion (*m*/*z*)	CE (eV) ^b^	Ion Mode	Content (µg/g)
1	Pyrogallol	7.32	124.86	69.31; 79.27	20; 23	Negative	ND
2	Gallic acid	9.46	169.70	80.50; 126.20	25; 16	Negative	ND
3	Protocatechuic acid	11.33	155.01	65.40; 93.20	22; 13	Positive	ND
4	Gentisic acid	11.33	153.70	109.50	21	Negative	ND
5	Protocatechuic aldehyde	12.12	136.90	92.25; 108.20	25; 25	Negative	ND
6	Sesamol	12.12	137.18	108.17; 109.29	15; 14	Negative	ND
7	Catechin	12.73	289.20	203.90; 245.70	22; 17	Negative	ND
8	Chlorogenic acid	13.79	353.40	86.50; 192.10	43; 21	Negative	871.43
9	Epicatechin	14.24	291.50	123.30; 139.30	15; 16	Positive	ND
10	Caffeic acid	14.44	179.70	135.20; 136.20	27; 18	Negative	ND
11	Vanillin	14.88	150.91	92.30; 136.10	23; 16	Negative	ND
12	Syringic aldehyde	15.42	180.88	151.09;166.09	22; 16	Negative	ND
13	Taxifolin	15.98	304.25	126.20; 285.50	23; 15	Negative	ND
14	*p*-coumaric acid	15.95	163.90	94.30; 120.20	33; 17	Negative	1599.71
15	Vitexin	16.43	430.90	283.03; 311.10	37; 24	Negative	ND
16	Ferulic acid	16.31	194.18	89.40; 177.40	30; 7	Positive	285.50
17	*p*-hydroxybenzoic acid	16.55	137.90	66.60; 94.60	38; 17	Negative	ND
18	Salicylic acid	16.65	137.14	65.50; 93.26	35; 18	Negative	39.72
19	Hesperidin	17.05	608.78	164.01; 301.34	32; 60	Negative	ND
20	Rosmarinic acid	17.14	359.18	134.30; 162.20	44; 20	Negative	ND
21	Oleuropein	17.14	539.10	275.80; 377.50	22; 16	Negative	ND
22	Syringic acid	17.20	198.17	77.30; 123.20	23; 13	Negative	ND
23	Luteolin-7-glucoside	17.50	446.89	284.00; 285.00	45; 40	Negative	0.50
24	Rutin	17.68	609.37	300.60; 301.70	38; 34	Negative	ND
25	Resveratrol	17.78	228.98	107.20; 135.10	22; 14	Positive	ND
26	Ellagic acid	18.82	301.67	174.15; 284.80	30; 34	Negative	ND
27	Naringenin	19.39	273.00	147.10; 153.00	20; 24	Positive	ND
28	Quercetin	19.88	301.00	152.10; 179.90	23; 20	Negative	ND
29	Luteolin	20.45	287.02	135.10; 153.00	32; 31	Positive	ND
30	Kaempferol	20.45	303.00	153.00; 165.00	33; 28	Positive	ND
31	Apigenin	21.38	268.86	117.19; 149.11	40; 27	Negative	ND
32	Pinocembrin	21.43	257.07	103.27; 153.11	34; 23	Positive	ND
33	Chrysin	22.85	254.97	103.28; 153.10	30; 33	Positive	ND
34	Galangin	22.89	268.85	169.15; 171.08	29; 31	Negative	ND
35	Flavone	23.20	222.90	77.28; 121.15	35; 26	Positive	ND

^a^ RT: Retention time, ^b^ CE: Collision energy. ND: Not detected under the applied LC–MS/MS conditions (below the limit of detection, LOD).

**Table 3:** Inhibitory activities of sample against 5-LOX *.

Sample and Standard	5-LOX Inhibition Activity IC_50_ (µg/mL)
SWP-Liq	33.05 ± 1.1 ^b^
INDO	10.74 ± 0.9 ^a^

* 5-LOX: 5-Lipoxygenase. SWP-Liq: pyrolysis-derived liquid fraction obtained from silkworm feeding waste. Values represent the mean ± SD of three technical replicate measurements (*n* = 3). Differences between two groups were evaluated using Student’s *t*-test within a column; different lowercase letters denote significance at *p* < 0.05.

**Table 4:** Inhibitory activities of sample against α-glucosidase *.

Sample and Standard	α-Glucosidase Inhibition Activity IC_50_ (µg/mL)
SWP-Liq	1682.3 ± 3.2 ^b^
Acarbose	272.0 ± 1.5 ^a^

* SWP-Liq: pyrolysis-derived liquid fraction obtained from silkworm feeding waste. Values represent the mean ± SD of three technical replicate measurements (*n* = 3). Differences between two groups were evaluated using Student’s *t*-test; within a column, different lowercase letters denote significance at *p* < 0.05.

**Table 5:** Determination of the antibacterial activity of the analyzed sample *.

	Diameter of Inhibition Zones, mm			
	Gram-Negative		Gram-Positive	
Samples	*E. coli*	*P. aeruginosa*	*S. aureus*	*E. faecalis*
SWP-Liq-1	13.6 ± 0.9 ^b^	17.2 ± 1.1 ^b^	17.4 ± 1.0 ^b^	12.5 ± 0.8 ^b^
SWP-Liq-2	NA	NA	NA	NA
Ampicillin	17.4 ± 1.2 ^a^	30.7 ± 1.5 ^a^	31.65 ± 1.3 ^a^	17.4 ± 1.2 ^a^

* SWP-Liq-1: 8 mg/mL, SWP-Liq-2: 4 mg/mL, and Ampicillin: 20 µg/mL. NA: Not active. Values represent the mean ± SD of three technical replicate measurements (*n* = 3); unequal superscript letters within a column denote significant pairwise differences by Tukey’s test (*p* < 0.05).

## Data Availability

The original contributions presented in this study are included in the article. Further inquiries can be directed to the corresponding author.

## References

[1] Dahiya S., Kumar A.N., Sravan J.S., Chatterjee S., Sarkar O., Mohan S.V. (2018). Food waste biorefinery: Sustainable strategy for circular bioeconomy. Bioresour. Technol..

[2] Koul B., Yakoob M., Shah M.P. (2022). Agricultural waste management strategies for environmental sustainability. Environ. Res..

[3] Lin C.S.K., Pfaltzgraff L.A., Herrero-Davila L., Mubofu E.B., Abderrahim S., Clark J.H., Koutinas A.A., Kopsahelis N., Stamatelatou K., Dickson F. (2013). Food waste as a valuable resource for the production of chemicals, materials and fuels. Current situation and global perspective. Energy Environ. Sci..

[4] Sadh P.K., Duhan S., Duhan J.S. (2018). Agro-industrial wastes and their utilization using solid state fermentation: A review. Bioresour. Bioprocess..

[5] Mohan D., Pittman C.U., Steele P.H. (2006). Pyrolysis of wood/biomass for bio-oil: A critical review. Energy Fuels.

[6] Goularte M.d.P., de Sousa Á.F., Cholant C., Romano L., Labidi J., Missio A.L., Acosta A., Gatto D.A. (2025). Pyrolysis of rice husk for the production of bioactive compounds with potential in green chemistry and sustainable agriculture. Molecules.

[7] Aznar-Cervantes S.D., Monteagudo Santesteban B., Cenis J.L. (2021). Products of sericulture and their hypoglycemic action evaluated by using the silkworm, *Bombyx mori* (Lepidoptera: Bombycidae), as a model. Insects.

[8] Fan M., Choi Y.-J., Wedamulla N.E., Zhang Q., Kim S.W., Bae S.M., Seok Y.-S., Kim E.-K. (2023). Use of a silkworm (*Bombyx mori*) larvae by-product for the treatment of atopic dermatitis: Inhibition of NF-κB nuclear translocation and MAPK signaling. Nutrients.

[9] Biganeh H., Kabiri M., Zeynalpourfattahi Y., Brancalhão R.M.C., Karimi M., Ardekani M.R.S., Rahimi R. (2022). *Bombyx mori* cocoon as a promising pharmacological agent: A review of ethnopharmacology, chemistry, and biological activities. Heliyon.

[10] Sadat A., Biswas T., Cardoso M.H., Mondal R., Ghosh A., Dam P., Nesa J., Chakraborty J., Bhattacharjya D., Franco O.L. (2022). Silkworm pupae as a future food with nutritional and medicinal benefits. Curr. Opin. Food Sci..

[11] Chen J., Gou Z., Huang Y., Yu Q., Kim A.N., Shi W., Zhou Y. (2025). Research progress on phytochemicals from mulberry with neuroprotective effects: A review. Pharmaceuticals.

[12] Farokhi M., Mottaghitalab F., Fatahi Y., Khademhosseini A., Kaplan D.L. (2018). Overview of silk fibroin use in wound dressings. Trends Biotechnol..

[13] Reddy N., Aramwit P. (2021). Sustainable Uses of Byproducts from Silk Processing.

[14] Ghosh A., Ray M., Gangopadhyay D. (2020). Evaluation of proximate composition and antioxidant properties in silk-industrial byproduct. LWT.

[15] Cermeno M., Bascón C., Amigo-Benavent M., Felix M., FitzGerald R.J. (2022). Identification of peptides from edible silkworm pupae (*Bombyx mori*) protein hydrolysates with antioxidant activity. J. Funct. Foods.

[16] Azmir J., Zaidul I.S.M., Rahman M.M., Sharif K.M., Mohamed A., Sahena F., Jahurul M.H.A., Ghafoor K., Norulaini N.A.N., Omar A.K.M. (2013). Techniques for extraction of bioactive compounds from plant materials: A review. J. Food Eng..

[17] Chemat F., Abert Vian M., Ravi H.K., Khadhraoui B., Hilali S., Perino S., Fabiano-Tixier A.S. (2019). Review of alternative solvents for green extraction of food and natural products: Panorama, principles, applications and prospects. Molecules.

[18] Bridgwater A.V. (2012). Review of fast pyrolysis of biomass and product upgrading. Biomass Bioenergy.

[19] Yaashikaa P.R., Kumar P.S., Varjani S., Saravanan A. (2020). A critical review on the biochar production techniques, characterization, stability and applications for circular bioeconomy. Biotechnol. Rep..

[20] Jerzak W., Acha E., Li B. (2024). Comprehensive review of biomass pyrolysis: Conventional and advanced technologies, reactor designs, product compositions and yields, and techno-economic analysis. Energies.

[21] Nadarajah K., Asharp T., Jeganathan Y. (2024). Biochar from waste biomass, its fundamentals, engineering aspects, and potential applications: An overview. Water Sci. Technol..

[22] Kaewkod T., Kumseewai P., Suriyaprom S., Intachaisri V., Cheepchirasuk N., Tragoolpua Y. (2024). Potential therapeutic agents of *Bombyx mori* silk cocoon extracts from agricultural product for inhibition of skin pathogenic bacteria and free radicals. PeerJ.

[23] Barbero-López A., Chibily S., Tomppo L., Salami A., Ancin-Murguzur F.J., Venäläinen M., Lappalainen R., Haapala A. (2019). Pyrolysis distillates from tree bark and fibre hemp inhibit the growth of wood-decaying fungi. Ind. Crops Prod..

[24] Özgen İ., Gül A., Baltacı C., Öner İ., Aydoğmuş E. (2025). Determination of antibacterial properties and chemical composition of pyrolysis liquid from feeding wastes of mealworm *Tenebrio molitor* L. (Coleoptera: Tenebrionidae) adults and larvae. J. Serb. Chem. Soc..

[25] Li S., Chen G. (2018). Thermogravimetric, thermochemical, and infrared spectral characterization of feedstocks and biochar derived at different pyrolysis temperatures. Waste Manag..

[26] Sun J., He F., Pan Y., Zhang Z. (2017). Effects of pyrolysis temperature and residence time on physicochemical properties of different biochar types. Acta Agric. Scand. B Soil Plant Sci..

[27] Kasangana P.B., Haddad P.S., Stevanovic T. (2015). Study of polyphenol content and antioxidant capacity of *Myrianthus arboreus* (Cecropiaceae) root bark extracts. Antioxidants.

[28] Gülmez G., Şen A., Servi H., Barak T.H., Tekin F., Marzi M., Şener A. (2025). A comparative study of biochemical, antimicrobial effects and phytochemical composition analysis of *Glycyrrhiza glabra* L. varieties root extracts. J. Res. Pharm..

[29] Yilmaz M.A. (2020). Simultaneous quantitative screening of 53 phytochemicals in 33 species of medicinal and aromatic plants: A detailed, robust and comprehensive LC–MS/MS method validation. Ind. Crops Prod..

[30] Ahmed D., Khan M.M., Saeed R. (2015). Comparative analysis of phenolics, flavonoids, and antioxidant and antibacterial potential of methanolic, hexanic and aqueous extracts from *Adiantum caudatum* leaves. Antioxidants.

[31] Oyaizu M. (1986). Studies on products of browning reaction: Antioxidative activities of products of browning reaction prepared from glucosamine. J. Jpn. Soc. Nutr. Food Sci..

[32] Apak R., Güçlü K., Özyürek M., Karademir S.E. (2004). Novel total antioxidant capacity index for dietary polyphenols and vitamins C and E, using their cupric ion reducing capability in the presence of neocuproine: CUPRAC method. J. Agric. Food Chem..

[33] Yıldırım A., Şen A., Doğan A., Bitis L. (2019). Antioxidant and anti-inflammatory activity of capitula, leaf and stem extracts of *Tanacetum cilicicum* (Boiss.) Grierson. Int. J. Second. Metab..

[34] Ramakrishna R., Sarkar D., Schwarz P., Shetty K. (2017). Phenolic linked anti-hyperglycemic bioactives of barley (*Hordeum vulgare* L.) cultivars as nutraceuticals targeting type 2 diabetes. Ind. Crops Prod..

[35] Kobya H.N., Gül A., Karpuz Ö., Baltacı C. (2026). Investigation of the Antioxidant, Antibacterial, and Food Safety Potential of Silver and Copper Nanoparticles Green Synthesized Using Pointed Cherry Laurel (*Prunus laurocerasus* L.) Extract. Food Bioprocess Technol..

[36] Wang W.H., Lin W.S., Shih C.H., Chen C.Y., Kuo S.H., Li W.L., Lin Y.S. (2021). Functionality of silk cocoon (*Bombyx mori* L.) sericin extracts obtained through high-temperature hydrothermal method. Materials.

[37] Wang H.Y., Zhao J.G., Zhang Y.Q. (2020). The flavonoid-rich ethanolic extract from the green cocoon shell of silkworm has excellent antioxidation, glucosidase inhibition, and cell protective effects in vitro. Food Nutr. Res..

[38] Zhou Y., Zhou S., Duan H., Wang J., Yan W. (2022). Silkworm pupae: A functional food with health benefits for humans. Foods.

[39] Guo Q., Jin Y., Chen X., Ye X., Shen X., Lin M., Zeng C., Zhou T., Zhang J. (2024). NF-κB in biology and targeted therapy: New insights and translational implications. Signal Transduct. Target. Ther..

[40] Mao H., Zhao X., Sun S.C. (2025). NF-κB in inflammation and cancer. Cell. Mol. Immunol..

[41] Salvemini D., Kim S.F., Mollace V. (2013). Reciprocal regulation of the nitric oxide and cyclooxygenase pathway in pathophysiology: Relevance and clinical implications. Am. J. Physiol. Regul. Integr. Comp. Physiol..

[42] Heo Y.J., Lee N., Choi S.-E., Jeon J.Y., Han S.J., Kim D.J., Kang Y., Lee K.W., Kim H.J. (2023). Amphiregulin induces iNOS and COX-2 expression through NF-κB and MAPK signaling in hepatic inflammation. Mediat. Inflamm..

[43] Chen M., Qin Y., Ma H., Zheng X., Zhou R., Sun S., Huang Y., Duan Q., Liu W., Wu P. (2019). Downregulating NF-κB signaling pathway with triterpenoids for attenuating inflammation: In vitro and in vivo studies. Food Funct..

[44] da Silva Júnior W.F., Pinheiro J.G.d.O., Moreira C.D.L.d.F.A., Rüdiger A.L., Barbosa E.G., Lima E.S., Júnior V.F.d.V., Júnior A.A.d.S., Aragão C.F.S., de Lima Á.A.N. (2017). Thermal behavior and thermal degradation kinetic parameters of triterpene α, β-amyrin. J. Therm. Anal. Calorim..

[45] Bakry S.M., El-Shiekh R.A., Hatem S., Mandour A.A., El-Dessouki A.M., Bishr A., Elosaily H., Mohamed A.F., Elhusseiny S.M. (2025). Therapeutic applications of ursolic acid: A comprehensive review and utilization of predictive tools. Future J. Pharm. Sci..

[46] Liu R., Zhang S., Tang Y., Li Q. (2025). Natural biocolourant selectivity from mulberry leaves to natural coloured cocoon silkworm excrement. Color. Technol..

[47] Chen F., Zhang X., Wang J., Wang F., Mao J. (2024). *p*-Coumaric acid: Advances in pharmacological research based on oxidative stress. Curr. Top. Med. Chem..

[48] Nguyen V., Taine E.G., Meng D., Cui T., Tan W. (2024). Chlorogenic acid: A systematic review on the biological functions, mechanistic actions, and therapeutic potentials. Nutrients.

[49] Purushothaman J.R., Rizwanullah M., Purushothaman R. (2024). Ferulic acid: A comprehensive review. Cureus.

[50] Liu D., Pan S., Sun J. (2025). Natural phenolic acids as promising antimicrobial candidates in food industry: A review. Int. J. Food Microbiol..

[51] Scarano A., Laddomada B., Blando F., De Santis S., Verna G., Chieppa M., Santino A. (2023). The chelating ability of plant polyphenols can affect iron homeostasis and gut microbiota. Antioxidants.

[52] Ahmad Z., Rauf A., Orhan I.E., Mubarak M.S., Akram Z., Islam R., Imran M., Edis Z., Kondapavuluri B.K., Thangavelu L. (2025). Antioxidant potential of polyphenolic compounds, sources, extraction, purification and characterization techniques: A focused review. Food Sci. Nutr..

[53] Zhu C., Lin Z., Jiang H., Wei F., Wu Y., Song L. (2024). Recent advances in the health benefits of phenolic acids in whole grains and the impact of processing techniques on phenolic acids: A comprehensive review. J. Agric. Food Chem..

[54] Sánchez-Maldonado A.F., Schieber A., Gänzle M.G. (2011). Structure–function relationships of the antibacterial activity of phenolic acids and their metabolism by lactic acid bacteria. J. Appl. Microbiol..

[55] Borges A., Ferreira C., Saavedra M.J., Simões M. (2013). Antibacterial activity and mode of action of ferulic and gallic acids against pathogenic bacteria. Microb. Drug Resist..

[56] Lou Z., Wang H., Rao S., Sun J., Ma C., Li J. (2012). *p*-Coumaric acid kills bacteria through dual damage mechanisms. Food Control.

[57] Naveed M., Hejazi V., Abbas M., Kamboh A.A., Khan G.J., Shumzaid M., Ahmad F., Babazadeh D., Xia F., Modarresi-Ghazani F. (2018). Chlorogenic acid (CGA): A pharmacological review and call for further research. Biomed. Pharmacother..

[58] Ansari K.B., Arora J.S., Chew J.W., Dauenhauer P.J., Mushrif S.H. (2019). Fast pyrolysis of cellulose, hemicellulose, and lignin: Effect of operating temperature on bio-oil yield and composition and insights into the intrinsic pyrolysis chemistry. Ind. Eng. Chem. Res..

[59] Stoenescu A.M., Stănică F. (2025). Comparative characterization of phenolic profile and antioxidant activity in homemade and commercial jujube vinegar. Beverages.

[60] Anuduang A., Loo Y.Y., Jomduang S., Lim S.J., Wan Mustapha W.A. (2020). Effect of thermal processing on physico-chemical and antioxidant properties in mulberry silkworm (*Bombyx mori* L.) powder. Foods.

[61] Chukiatsiri S., Wongsrangsap N., Kiatwuthinon P., Phonphoem W. (2024). Purification and identification of novel antioxidant peptides derived from *Bombyx mori* pupae hydrolysates. Biochem. Biophys. Rep..

[62] Loo A.Y., Jain K., Darah I. (2008). Antioxidant activity of compounds isolated from the pyroligneous acid, *Rhizophora apiculata*. Food Chem..

[63] Fan J.B., Wu L.P., Chen L.S., Mao X.Y., Ren F.Z. (2009). Antioxidant activities of silk sericin from silkworm *Bombyx mori*. J. Food Biochem..

[64] Knez E., Kadac-Czapska K., Grembecka M. (2025). Evaluation of spectrophotometric methods for assessing antioxidant potential in plant food samples—A critical approach. Appl. Sci..

[65] Yuan Q., Liu C., Zhang Z., Chen F., Xiao Q., Zhang L., Pan X., He F., Xiao M. (2025). Pharmacological advances of the chlorogenic acids family: Current insights and future research directions. Front. Pharmacol..

[66] Zhou Y., Wang D., Guo J., Zheng Y., Duan H., Liu G., Yan W. (2024). Silkworm pupa protein-derived peptides alleviate LPS-induced inflammatory response in RAW264.7 macrophage cells through the NF-κB/MAPK/PI3K-AKT signaling pathway. J. Agric. Food Res..

[67] Kim S., Hong S., Choi K., Kim S., Jeong S., Park S. (2019). Antibacterial and anti-inflammatory activities of the immune-challenged silkworm (*Bombyx mori*) hemolymph with *Lactobacillus* cell wall extracts. Entomol. Res..

[68] Kim D., Hwang H., Kim D., Sheen S., Heo D., Hwang G., Kang S., Kweon H., Jo Y., Kang S. (2011). Effect of silk fibroin peptide derived from silkworm *Bombyx mori* on the anti-inflammatory effect of Tat-SOD in a mice edema model. BMB Rep..

[69] Kyriaki A., Vidali M., Vitsos A., Harizanis P., Sfiniadakis I., Barda C., Simirioti E., Terezaki A., Rallis M. (2024). Comparative evaluation of wound healing efficacy of *Bombyx mori* L. body extracts, gland extracts, and cocoon for the treatment of second-degree burns: A pilot study. Processes.

[70] Alssema M., Ruijgrok C., Blaak E.E., Egli L., Dussort P., Vinoy S., Dekker J.M., Robertson M.D. (2021). Effects of alpha-glucosidase-inhibiting drugs on acute postprandial glucose and insulin responses: A systematic review and meta-analysis. Eur. J. Clin. Nutr..

[71] Wang H., Wang Y., Zhou L., Zhu L., Zhang Y. (2012). Isolation and bioactivities of a non-sericin component from cocoon shell silk sericin of the silkworm *Bombyx mori*. Food Funct..

[72] Zhao J., Wang H., Wei Z., Zhang Y. (2019). Therapeutic effects of ethanolic extract from the green cocoon shell of silkworm *Bombyx mori* on type 2 diabetic mice and its hypoglycaemic mechanism. Toxicol. Res..

[73] Rattana S., Katisart T., Butiman C., Sungthong B. (2017). Antihyperglycemic effect of silkworm powder, fibroin and sericin from three Thai silkworm (*Bombyx mori* Linn.) in streptozotocin-induced diabetic rats. Pharmacogn. J..

[74] Rodríguez-Ortiz L., Hincapié C., Hincapié-Llanos G., Osorio M. (2024). Potential uses of silkworm pupae (*Bombyx mori* L.) in food, feed, and other industries: A systematic review. Front. Insect Sci..

[75] Chirila T., Suzuki S., McKirdy N. (2016). Further development of silk sericin as a biomaterial: Comparative investigation of the procedures for its isolation from *Bombyx mori* silk cocoons. Prog. Biomater..

[76] Nikaido H. (2003). Molecular basis of bacterial outer membrane permeability revisited. Microbiol. Mol. Biol. Rev..

[77] Patra J.K., Hwang H., Choi J.W., Baek K.H. (2015). Bactericidal mechanism of bio-oil obtained from fast pyrolysis of *Pinus densiflora* against two foodborne pathogens, *Bacillus cereus* and *Listeria monocytogenes*. Foodborne Pathog. Dis..

